# Teaching Everyone Everywhere All at Once: Leveraging Social Media to Implement a Multisite Fungal Diagnostics Curriculum

**DOI:** 10.1093/ofid/ofad594

**Published:** 2023-11-22

**Authors:** Saman Nematollahi, Sean Tackett, Suzanne Grieb, Justin C Laracy, Anne Belcher, Kieren A Marr, Shmuel Shoham, Robin K Avery, Michael T Melia

**Affiliations:** Department of Medicine, University of Arizona College of Medicine, Tucson, Arizona, USA; Johns Hopkins Bayview Medical Center, Johns Hopkins University School of Medicine, Baltimore, Maryland, USA; Biostatistics, Epidemiology, and Data Management Core, Johns Hopkins School of Medicine, Baltimore, Maryland, USA; Biostatistics, Epidemiology, and Data Management Core, Johns Hopkins School of Medicine, Baltimore, Maryland, USA; Department of Pediatrics, Center for Child and Community Health Research, Johns Hopkins School of Medicine, Baltimore, Maryland, USA; Department of Medicine, Memorial Sloan Kettering Cancer Center, New York, New York, USA; Department of Medicine, Weill Cornell Medical College, New York, New York, USA; Johns Hopkins University School of Education, Baltimore, Maryland, USA; Department of Medicine, Johns Hopkins University School of Medicine, Baltimore, Maryland, USA; Pearl Diagnostics, Baltimore, Maryland, USA; Department of Medicine, Johns Hopkins University School of Medicine, Baltimore, Maryland, USA; Department of Medicine, Johns Hopkins University School of Medicine, Baltimore, Maryland, USA; Department of Medicine, Johns Hopkins University School of Medicine, Baltimore, Maryland, USA

**Keywords:** fungal diagnostics, infectious diseases, medical education, social media, virtual learning

## Abstract

**Background:**

Environmental fungi are threats to personal and public health. Fungal in vitro diagnostics help diagnose invasive fungal infections (IFIs), but clinicians remain underinformed about their use and interpretation. Given the increasing use of social media to share infectious diseases–related content, we designed and implemented a multisite Twitter-based curriculum focused on IFIs and related diagnostics.

**Methods:**

Questions were posted through a dedicated Twitter account twice weekly over 8 weeks. We surveyed clinicians at 3 US academic centers before and after completion of the curriculum and interviewed a subset of participants. We undertook quantitative and qualitative evaluations and reviewed Twitter analytics.

**Results:**

We surveyed 450 participants. One hundred twenty-one participants (27%) completed the knowledge assessment precurriculum, 68 (15%) postcurriculum, and 53 (12%) pre- and postcurriculum. We found a significant increase (72% vs 80%, *P* = .005) in the percentage of correct answers in the pre- versus postcurriculum knowledge assessments. Perceived benefits included a well-executed curriculum that facilitated engagement with appropriately detailed tweetorials from a dedicated Twitter account. Perceived barriers included lack of awareness of tweetorial posts and timing, competing priorities, and the coronavirus disease 2019 pandemic. The Twitter account accrued 1400 followers from 65 countries during the 8-week period. Tweets with multiple-choice questions had a median of 14 904 impressions (interquartile range [IQR], 12 818–16 963), 798 engagements (IQR, 626–1041), and an engagement rate of 6.1% (IQR, 4.2%–6.6%).

**Conclusions:**

Educators can leverage social media to share content with a large audience and improve knowledge while being mindful of the barriers associated with implementing a curriculum on social media.

## Teaser

We implemented a multisite Twitter-based curriculum focused on fungal infections and diagnostics. We demonstrated a wide dissemination of content and observed a significant increase in knowledge. This article highlights ways in which educators can leverage social media to improve knowledge.

Environmental fungi are ubiquitous and can cause invasive infections in both immunocompetent and immunocompromised hosts. Fungi are increasingly recognized as important threats to public health on a global level as evidenced by the World Health Organization's recently published fungal pathogen priority list [1]. Given the limited sensitivity of culture-based diagnostics, in vitro diagnostics that detect cellular antigens, especially galactomannan (GM) and β-d-glucan (BDG), are now commonly used to aid in diagnoses of invasive fungal infections (IFIs). The clinical utility of in vitro diagnostics varies according to factors that impact analytic performance (eg, circulating fungal burden) and predictive value (eg, host conditions that impact prevalence of infection). The serum Platelia GM enzyme immunoassay is cleared as an aid to diagnose invasive aspergillosis, with best performance in people with high risks associated with neutropenia [2]. The Fungitell BDG assay is less specific and cleared as an aid to diagnose IFIs in people with high risks; the assay cutoff for positivity was generated in people with highest risks for candidemia [3].

Despite studies describing analytic and clinical performance, and widespread increased understandings of IFI risks, clinicians remain poorly informed regarding appropriate use and interpretation of these tests. A prior knowledge assessment among internal medicine (IM) residents at 1 academic center demonstrated poor knowledge of BDG and GM test characteristics [4]. Among nonneutropenic patients at this center, 49% of BDG orders were deemed inappropriate due to lack of risk factors for or absence of a clinical syndrome indicative of an IFI, and most of the inappropriate BDG ordering was performed by IM services [4]. Inappropriate and nonuse of fungal diagnostics can lead to overdiagnosis and missed diagnoses of IFIs, respectively, which can negatively impact patient outcomes.

Over the past several years, medical educators have increasingly used social media platforms such as Twitter to spread knowledge [5, 6] without the time and space constraints of traditional classrooms. Specifically, Twitter has been used to engage learners and disseminate information via multiple-choice questions followed by “tweetorials” (strings of tweets that provide educator-driven content centered around a chosen topic) [7, 8]. Although Twitter has been shown to increase medical knowledge [9, 10], it has not been incorporated into a multisite study. The overarching goal of this program was to design, implement, and evaluate a novel, multisite Twitter-based curriculum focused on IFI and related diagnostics.

## METHODS

### Setting and Participants

We offered the curriculum to IM residents, hospitalists, advanced practice providers, and intensive care unit (ICU) providers (hereafter, all participants will collectively be referred to as “providers”) at the Johns Hopkins Hospital (JHH), Johns Hopkins Bayview Medical Center (Bayview), and Columbia University Medical Center. One of the authors (S. N.) spoke at residency program didactic conferences and at ICU and hospital medicine faculty and staff meetings to inform prospective participants about the program and encourage them to create a Twitter account. Emails were subsequently sent to each group to remind potential participants about the curriculum. This project was approved by the Johns Hopkins and Columbia University institutional review boards (IRB00237977 and IRBAAAT2318, respectively).

### Twitter-Based Curriculum

**Figure 1.**
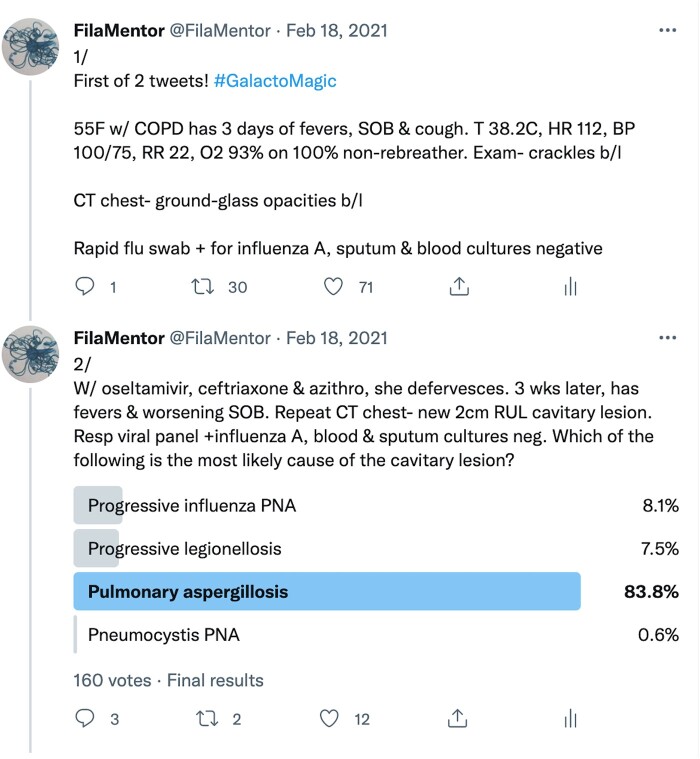
First 2 tweets of a tweetorial showing a multiple-choice question.

We selected topics and developed learning objectives based on a prior study showing limited knowledge of BDG and GM characteristics and frequent inappropriate BDG ordering despite the absence of risk factors for a syndrome suggestive of an IFI [4]. Twice weekly over 8 weeks (2 February–25 March 2021), we posted 16 clinical vignette–based, multiple-choice questions focused on fungal diagnostics, interpretation of BDG and GM results, and risk factors for IFI on Twitter (Figure 1). Each question was linked to at least 1 learning objective from our test blueprint (Supplementary Material A). Twenty-four hours after each question was posted, a relevant tweetorial was released. Each tweetorial spanned 10–15 tweets. Topics included reasons for false-positive BDG, indications for serum BDG and GM, risk factors for IFIs, evaluation of a solitary pulmonary nodule, and specific fungal infections (mucormycosis, histoplasmosis, coccidioidomycosis, cryptococcosis, *Pneumocystis* pneumonia, and aspergillosis). A new Twitter handle (@FilaMentor) was created for this program and was used only for this project. Author S. N. (who had approximately 4000 followers at the time of program initiation) retweeted all of the multiple-choice questions from his personal Twitter handle. The tweetorials were written by S. N. and reviewed by M. T. M.

### Evaluation

Our evaluation consisted of pre- and postcurriculum surveys of intended participants, review of Twitter analytics data, and 1-on-1 interviews with residents in the medical education pathway.

#### Pre–Post Surveys

We created pre- and post-Twitter curriculum surveys by following survey development guidelines [11]. In the surveys (Supplementary Material B), we collected demographic and background data (including prior IFI education, prior experience with fungal diagnostics, and preferred method of educational content delivery), assessed knowledge, and inquired about the quality and content of the Twitter-based questions and tweetorials.

The knowledge assessment portion of the surveys comprised 10 clinical vignette–based multiple-choice questions. The same learning objectives were addressed on each of the 2 surveys; the clinical vignette stems were unique. To provide content validity, a panel with expertise in diagnostic stewardship, medical education, and fungal infections (K. A. M., R. K. A., S. S., and M. T. M.) reviewed the questions. The surveys were piloted with chief medical residents and infectious diseases (ID) fellows at participating sites. We performed 1-on-1 cognitive interviews with 3 IM residents and ID fellows to assess prospective respondents’ interpretation of survey items. We adjusted the surveys based upon pilot and interview feedback.

The directors of the IM residency programs provided us with a list of all residents. The directors of the medical ICU and hospital medicine programs provided us with a list of providers. Using these lists and Qualtrics software, we emailed a link to the prevignette survey to providers at each site. Weekly participation reminders were sent for 4 weeks. One week after sending the final prevignette survey reminder, we launched the Twitter vignette curriculum. One week after the final Twitter vignette and tweetorial were posted, we used Qualtrics software to email a link of the postvignette survey to providers at each site. Weekly participation reminders were sent for 4 weeks. Participation was voluntary and incentivized with a gift card lottery.

#### Twitter Analytics

Twitter analytics for the @FilaMentor account were obtained using the Twitter website and Tweepsmap. Specifically, 4 weeks after the final question was released, we measured impressions (total number of times users saw each tweet), engagement (total number of times a user interacted with each tweet), and engagement rate (number of engagements divided by number of impressions for each tweet). Since Twitter is a public social media platform, we were unable to collect engagement data at the individual participant level.

#### Interviews

To augment the information provided by the surveys, after completion of the postvignette surveys, we emailed the 24 residents in the medical education pathway within the JHH and Bayview IM residency programs to solicit participation in a 30- to 60-minute, 1-on-1 interview. Topics included perceived benefits and barriers to curriculum participation and ways to increase participation in Twitter-based education projects.

### Data Analysis

We summarized survey responses with descriptive statistics. For learning objectives, we calculated a total mean for each participant across all items relevant for that learning objective. We used paired statistical tests (eg, paired *t* tests, Wilcoxon signed-rank test, McNemar test) for pre–post items as appropriate. All *P* values were 2-sided, and a result of ≤.05 was considered significant. Analyses were done using Stata version 13.0 software (StataCorp, College Station, Texas). Data visualization was performed using ggplot2 and dplyr using R statistical software (version 4.0.3) [12–14].

All interviews were audio-recorded and transcribed in their entirety. An iterative, constant comparative approach was used to analyze all interview data [15]. The initial codebook was edited after review and discussion with the interviewer (S. N.). Two qualitative coders systematically applied the codes to the transcripts in Atlas.ti software and conducted a thematic analysis [16]. Code output was synthesized to develop the final thematic structure [15, 17]. To improve reporting accuracy, all qualitative data are presented using direct quotes.

## RESULTS

### Setting and Participants

The pre- and postvignette surveys were sent to 450 participants (154 JHH residents, 142 Columbia residents, 64 Bayview residents, 38 JHH ICU providers, 34 JHH hospitalists, 17 Bayview hospitalists, 1 Columbia ICU provider). Table 1 shows participants’ training level and institution. One hundred twenty-one participants (27%) completed the knowledge assessment precurriculum, 68 (15%) postcurriculum, and 53 (12%) pre- and postcurriculum. Of the 121 participants, 29 (24%) reported never having used Twitter. With respect to the preferred method of educational content delivery, the most preferred method was social media, followed by self-paced online modules, small group sessions, and journal articles.

**Table 1.** Pre- and Postvignette Survey Respondent Demographics

Characteristic	Presurvey (n = 121)	Postsurvey (n = 68)
	Respondents, No. (%^a^)	Respondents, No. (%)
Position		
PGY-1	30 (25)	18 (27)
PGY-2	39 (32)	25 (37)
PGY-3	29 (24)	13 (19)
PGY-4	1 (1)	1 (1)
Hospitalist	10 (8)	5 (7)
APP	3 (3)	2 (3)
ICU attending	9 (7)	4 (6)
Total	121 (27^b^)	68 (15^b^)
Institution		
Columbia	39 (32)	15 (22)
JHH	63 (52)	37 (54)
Bayview	19 (16)	16 (24)
Total	121 (27^b^)	68 (15^b^)

Abbreviations: APP, advanced practice provider; ICU, intensive care unit; JHH, Johns Hopkins Hospital; PGY, postgraduate year.

^a^Percentage of all 121 respondents.

^b^Percentage of all 450 invited participants.

Twelve (50%) medical education pathway residents (5 postgraduate year [PGY]-1, 3 PGY-2, 3 PGY-3, and 1 PGY-4) completed in-depth interviews (5 from JHH, 7 from Bayview). All interview participants were aware of the curriculum, and all but 1 participated in some curriculum activities.

### Surveys: Confidence and Knowledge

**Figure 2.**
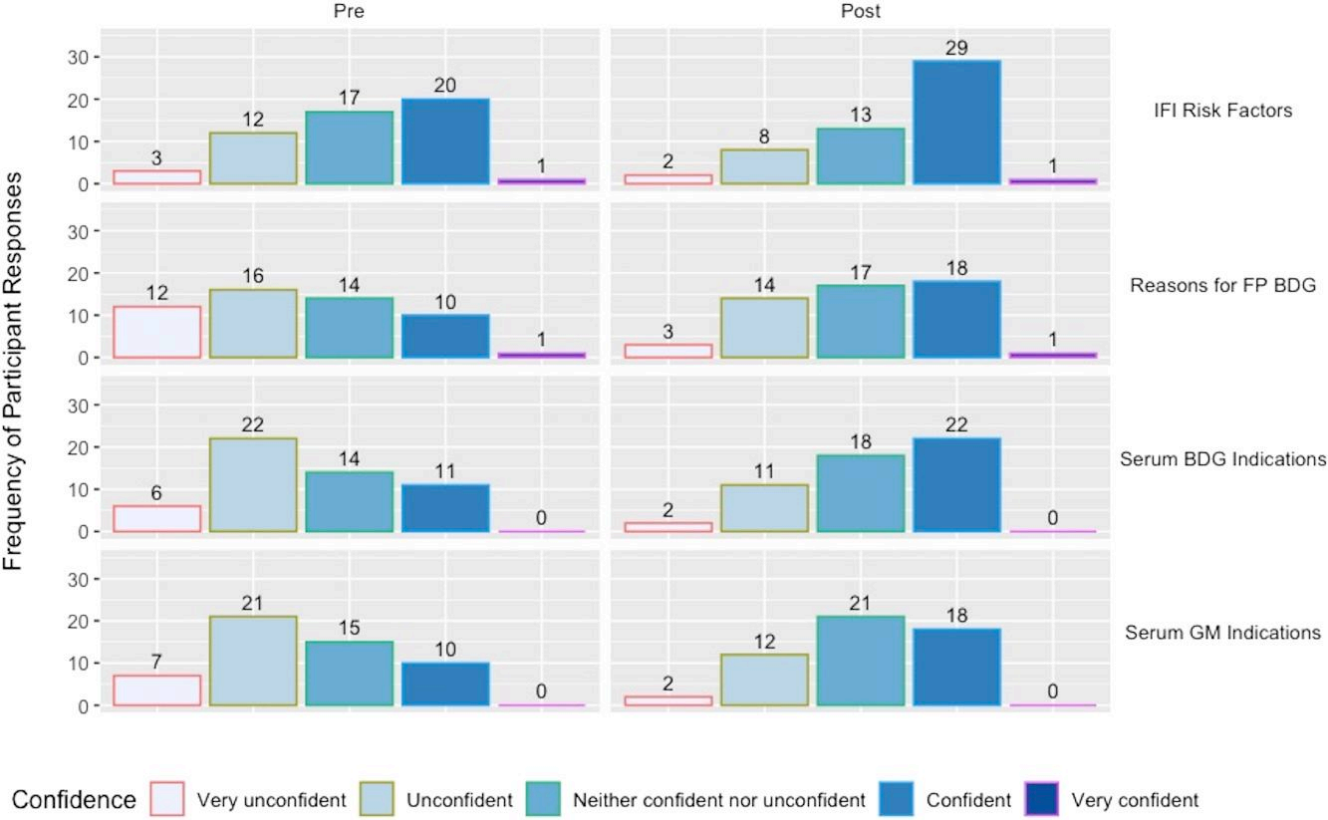
Participant confidence pre- and postcurriculum. Abbreviations: BDG, β-d-glucan; FP, false positive; GM, galactomannan; IFI, invasive fungal infection.

The majority (47/53 [89%]) of respondents felt that having knowledge of IFI risk factors and test characteristics of BDG and GM was either important or very important to their clinical practice. Comparing the pre- to postvignette survey responses, participants reported significant increases in confidence in their ability to name IFI risk factors, indications for serum BDG, indications for serum GM, and reasons for a false-positive serum BDG (*P* < .05; Figure 2). We found a significant increase in total knowledge assessment scores (72% vs 80%; *P* = .005) (Table 2). Knowledge assessment scores increased for each of the individual learning objectives; these increases were significant for identification of appropriate indications for BDG and GM testing, respectively (Table 2).

**Table 2.** Survey Respondent Knowledge Assessment

Learning Objective	Presurvey (n = 51)			Postsurvey (n = 51)			*P* Value
	Mean	SD	Range	Mean	SD	Range	
Distinguish at least 1 risk factor for each of the following fungal infections	70%	18%	33%–100%	76%	18%	33%–100%	.071
Identify 1 cause of false positive of BDG testing^a^	75%	…	0%–100%	84%	…	0%–100%	.581
Identify 1 appropriate indication for BDG testing	58%	23%	0%–100%	75%	20%	25%–100%	.000
Identify 1 appropriate indication for GM testing	65%	23%	0%–100%	75%	20%	25%–100%	.007
Total	72%	16%	40%–100%	80%	14%	50%–100%	.005

Abbreviations: BDG, β-d-glucan; GM, galactomannan; SD, standard deviation.

^a^This learning objective had 1 test item.

### Surveys: Engagement and Feedback

In the postvignette survey, 10% (7/68) of respondents stated that they completed >50% of the Twitter clinical vignette–based questions. Eighty-two percent were satisfied or very satisfied with the quality of the Twitter questions, and 69% considered the Twitter questions to be difficult. Ninety percent believed that the content was clear, and 100% felt that the content was informative. Finally, 65% thought that Twitter was effective as a learning tool, and 65% would participate in a Twitter-based curriculum again.

### Twitter Analytics

**Figure 3.**
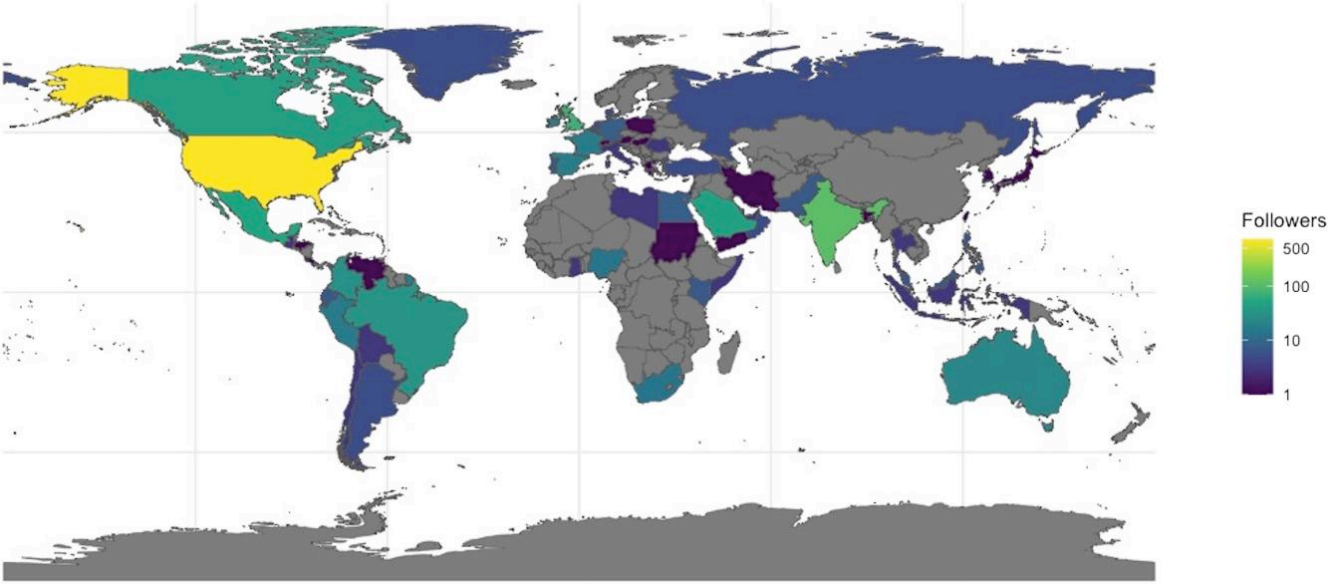
Global map showing @FilaMentor followers from 65 countries.

During the 8-week period, the @FilaMentor account accrued 1400 followers from 65 countries. The majority were from the United States (US) (53%), followed by India (7.9%), the United Kingdom (5.1%), Mexico (3.7%), Saudi Arabia (3.3%), and Canada (3.1%) (Figure 3). Within the US, the account had followers from >40 states, with the majority from Maryland (7.6%), followed by New York (6.4%) and California (5.2%). Tweets containing multiple-choice questions had a median of 14 904 impressions (interquartile range [IQR], 12 818–16 963) with 798 engagements (IQR, 626–1041), and an engagement rate of 6.1% (IQR, 4.2%–6.6%). Several tweets had higher metrics, with maximum values of 28 060 impressions, 1700 engagements, and a 9.5% engagement rate.

### Interviews

The perceived benefits of the curriculum included program characteristics that filled knowledge gaps and facilitated engagement (Table 3). Specifically, participants felt that the curriculum was well-executed with appropriately detailed tweetorials distributed through a dedicated Twitter account, and that the range of questions and topics would appeal to people with varied clinical interests.

**Table 3.** Themes and Subthemes Generated From the Resident Interviews

Themes	Subthemes	Quotes
Benefits of Twitter-based fungal diagnostics curriculum	There is a need for more information on this topic	“One other thing I should add is that I think that the topic is a good topic. I think all of us want to learn more about fungal infections, and there's a need for it, a need for this education. And so, I don't think that there's any lack of interest. If that makes sense. I think that people want to learn about this. And they're like, oh, this would be a great thing for me to know, like when to send these studies, when to have a high clinical suspicion for certain infections.” (PGY-1, JHH)
	Program characteristics facilitated engagement	“You touched on a lot of these fields, because I mean, ID is like whole body medicine, that interacts with all these fields. I think there was a case of someone with cirrhosis that had SBP and had a fungal infection etiology. And then I think there was transplant and oncology.” (PGY-1, Bayview)
Barriers to participation in Twitter-based fungal diagnostics curriculum	Tweetorial postawareness and timing	“I think one problem that I encountered is I wasn't sure how frequently they would come out. I think I would just kind of happen along upon them. And when I saw them, I would do them. But I don't know how many I may have missed. I feel like I definitely didn't do all of them” (PGY-1, Bayview)
	Competing priorities	“I was on MICU and CCU during that time, and so I wasn't checking Twitter that often. And then when I would check Twitter, I was like a couple questions behind until I tried to catch up. And then I couldn't.” (PGY-2, JHH)
	Implementations during the COVID-19 pandemic: survey fatigue	“I get so many emails and like so many surveys to fill out. I've honestly been really overwhelmed … and I stopped doing [them] because there are so many.” (PGY-1, JHH)
	Implementations during the COVID-19 pandemic: tech overload	“Now when everything is virtual, and so you're already using technology for most of it, tweetorials don't have that same interest, like new dynamic to it that would initially have people doing it as a supplement to the education they already have. So, I think just the technology overload that is happening, because everything is virtual. I think contributes to why people were like, oh, gosh, I'm already tired of staring at a screen all day. Like, I don't. I don't even use Twitter that much. I'm just like, I can't. I can't look at more screens to tell more information. So at least for me, that was a major thing.” (PGY-2, Bayview)
	Implementations during the COVID-19 pandemic: burnout	“I dealt with higher levels of burnout individually … Even my personal engagement and … things that I was doing regularly, all that definitely curbed. And the impact of COVID wasn't just enough for us who were in direct care, but it was that a lot of residents were in a small program. So, residents were calling out due to COVID concerns or quarantines or sore throats throughout the year. So, people were being called in on their elective rotations or their lighter clinical rotations to go into the hospital constantly. So COVID was a huge burden on us.” (PGY-3, Bayview)
	Implementations during the COVID-19 pandemic: inability to promote program in person	“COVID kind of limited number of ways you're able to advertise the house staff. I actually never had been part of in-person noon conference in Hopkins so far just because the intern year was all consumed by COVID. And we went to the Zoom-based teaching for noon conference, but maybe if we had in-person conference, you could have had an opportunity to kind of meet us in person to kind of advertise it directly to us. And probably most of the residency would have been there in-person, that would run to kind of hear about this curriculum. So, I think the way it was advertised could have been enhanced if COVID-19 wasn't a thing.” (PGY-1, JHH)

Abbreviations: CCU, cardiac care unit; COVID-19, coronavirus disease 2019; ID, infectious diseases; JHH, Johns Hopkins Hospital; MICU, medical intensive care unit; PGY, postgraduate year; SBP, spontaneous bacterial peritonitis.

There were several perceived barriers to participation in the curriculum (Table 3). One barrier was lack of awareness about tweetorial posts and uncertainty regarding clinical vignette and tweetorial timing. Another barrier was competing priorities, such as busy clinical rotations and boards examination preparation. Finally, implementing a Twitter-based curriculum introduced pandemic-driven issues of survey fatigue, tech overload, burnout, and an inability to promote the program in person.

## DISCUSSION

We herein describe a multisite Twitter-based curriculum focused on fungal infections and diagnostics. Most notably, despite limited promotion of the curriculum outside of the intended participants, there was widespread dissemination of content both nationally and internationally. Comparing the pre- to postvignette survey responses, we found a significant increase in knowledge assessment scores. Participants enjoyed the content and reported that they would participate in a Twitter curriculum again. Finally, participants noted several barriers to participation that warrant reflection when designing and evaluating Twitter-based medical education curricula.

Few data have been published regarding the implementation of ID-related social media curricula. In a single-center study, investigators used both Twitter and Facebook to design an antimicrobial stewardship curriculum focused on creating awareness of resources and improving knowledge. Questions were posted on both platforms followed by published manuscripts or links to internal resources. Among IM resident participants, pre- to postcurriculum knowledge assessment test scores increased significantly (from 60% to 65%) [18]. In our program, we also noted an increase in test scores, and participants felt that the program characteristics facilitated engagement. Although we noticed an increase in test scores across all 4 learning objectives, the increases were significant for identification of appropriate indications for BDG and GM testing. This could be due to several reasons, including greater knowledge of certain topics, such as IFI risk factors or reasons for false-positive BDG, before the curriculum (as evidenced by higher presurvey scores compared to the other 2 learning objectives) with an associated ceiling effect; differential levels of difficulty for multiple-choice questions addressing different learning objectives; differential levels of Twitter curriculum effectiveness in teaching different topics; and/or that participants who completed both the pre- and postcurriculum surveys may have missed parts of the curriculum that addressed some of the learning objectives. Our findings comport with other descriptions of the medical education benefits of Twitter, including the ways in which it allows for interactivity and social learning [19]. Other learning strategies fostered by Twitter that may have been associated with our observed increase in test scores and program engagement include its use of technology-enhanced learning [20], self-directed learning [21], and cognitive learning strategies such as generation and elaboration, spaced retrieval, interleaving, and reflection [22].

Rates of engagement with our questions and tweetorials were high, with a median engagement rate of 6.1%. While there are no published data regarding mean or median rates of engagement with medical education content on social media, according to the 2023 Social Media Industry Benchmark Report, the median engagement rate across all industries is 0.037% [23]. Combined with our other Twitter analytics data, these data demonstrate that despite the low participation rate across our 3 sites, our curriculum found widespread national and international audiences. Moreover, 65% of participants thought that Twitter was effective as a learning tool and 65% would participate in a Twitter-based curriculum again, suggesting that a Twitter-based curriculum could supplement traditional education methods. It is further notable that the reach of our focused ID curriculum was achieved without concerted promotion of a previously unused Twitter handle, reflecting the desire for this ID-related content on social media. Engaging learners with ID-related topics on social media may represent a novel approach to engage early-stage learners who are interested in the field of ID and to expand awareness of the specialty.

Although participants felt our curriculum was well-done and facilitated engagement, we encountered substantial barriers to implementing and studying our curriculum during the coronavirus disease 2019 (COVID-19) pandemic. At all 3 sites, IM residents reported feeling inundated with weekly surveys. Concern for survey fatigue among graduate medical education (GME) trainees is not a new problem. In a qualitative study of 33 GME trainees, negative emotions such as annoyance and survey fatigue were identified as barriers to completing surveys. The trainees noted that a high volume of survey invitations and emails played a role in their low response rates [24]. Other studies provide additional evidence of low response rates to surveys due to clinical demands, the high burden of surveys, and survey fatigue [25–27]. Given that many medical education studies rely heavily on survey data, it is crucial for education stakeholders to address this problem.

Consistent with data from other reports, the topic of burnout during COVID-19 was mentioned several times in the interviews. Among 300 residents who responded to a survey about stress and burnout during the COVID-19 pandemic, 27% reported high stress, 30% reported anxiety and work overload, and >50% reported symptoms of burnout [28]. While efforts to improve physician well-being have been well-articulated in other reports [29, 30], from the perspective of our project, burnout diminished motivation to participate in our curriculum, especially in the background of survey fatigue and other virtual educational activities.

Given the limited experience and published guidance, we wish to provide guidance to educators who aim to implement longitudinal curricula on social media. First, support from key stakeholders to allot dedicated time to learners to complete questions and read tweetorials will help ensure the curriculum's success. To help facilitate this, educators may wish to carve out the beginning portion of time set aside for education (eg, morning report) to allow trainees to complete the question and tweetorial. This need not happen every day for a given curriculum. For example, when we asked participants how often they prefer to receive Twitter questions and tweetorials, the most common response was once a week. Second, to ensure that trainees are aware of the question and tweetorial posts, educators may wish to create a unique Twitter account dedicated exclusively to the educational content, and they may wish to inform learners how to follow and turn on tweet notifications for that specific account. Third, implementing a virtual curriculum with limited social interaction is challenging. Interviewees highlighted the value of in-person education and program promotion. Even outside of a pandemic, if feasible, to improve participation and engagement in a longitudinal Twitter-based curriculum, educators may wish to dedicate a portion of a highly attended conference to introducing the curriculum and teaching residents how to use Twitter and set up relevant notifications. Educators may also wish to meet trainees on the wards or in work rooms to promote the curriculum rather than advertising it exclusively virtually. These measures may help participants feel more connected as part of a collective learning experience to optimize learner engagement. For educators who wish to explore Twitter content with learners or address misinformation/disinformation on social media, we suggest relevant review articles [19, 31].

This study has some limitations. One limitation is the low response rate and potential for selection bias. Second, because of our low response rate, we were unable to meaningfully stratify our results by PGY level, site, or provider level. Third, although our observed improvement in knowledge assessment scores was statistically significant, an increase from 72% to 80% may not be educationally significant. Fourth, Twitter does not facilitate analysis of question responses at an individual level, which prevented us from conducting a correlation analysis between the performance on Twitter questions and the Qualtrics survey questions. Fifth, although the multisite program is a strength, there was variability in the environment and culture at each site with respect to social media presence, timing of additional surveys, and rates of SARS-CoV-2 infections. Sixth, this study measured the knowledge impact of the curriculum at 3 US institutions and may not reflect its impact at other US or international sites. Finally, although Twitter is a rapidly evolving platform, we feel that our approach may be used for similar emerging platforms. Two additional possible future avenues of study include assessing the durability of knowledge retention from a social media curriculum and incorporating measurement of impact on clinical practice.

## CONCLUSIONS

In this program, we implemented a multisite Twitter-based curriculum focused on fungal infections and diagnostics. The strengths of this project include its multisite design, mixed-methods evaluation, use of a novel Twitter handle to permit assessment of reach of the curriculum, and its international reach. We demonstrated a wide dissemination of content over social media and observed absolute increases in knowledge across all 4 learning objectives, with significant increases in 2 of these. Our data highlight ways in which educators can leverage social media to share content with a large audience to improve knowledge, especially in a time where virtual learning is here to stay.

## Supplementary Material

ofad594_Supplementary_Data

## Supplementary Data

Supplementary materials are available at *Open Forum Infectious Diseases* online. Consisting of data provided by the authors to benefit the reader, the posted materials are not copyedited and are the sole responsibility of the authors, so questions or comments should be addressed to the corresponding author.

## Notes

***Acknowledgments***. We would like to thank Dr Amy Nematollahi for data visualization; Drs Valeria Fabre and Sara Cosgrove for review of the initial grant and study design; the Johns Hopkins University Master of Education in the Health Professions for support while S. N. was in the master's program; and the internal medicine residency programs of Johns Hopkins Hospital, Johns Hopkins Bayview, and Columbia University for their participation and promotion of the study.

***Patient consent*.** This study does not include factors necessitating patient consent.

***Financial support***. This work was supported by the Sherrilyn and Ken Fisher Center for Environmental Infectious Diseases, Division of Infectious Diseases of the Johns Hopkins University School of Medicine.
