# Supplementary material for: Teaching Everyone Everywhere All at Once: Leveraging Social Media to Implement a Multisite Fungal Diagnostics Curriculum
Source: Open Forum Infect Dis. 2023 Nov 22;10(12):ofad594. doi: 10.1093/ofid/ofad594 (PMC10715681; doi:10.1093/ofid/ofad594)
Supplement: ofad594_Supplementary_Data [file ofad594_supplementary_data.docx]

**Supplement A: Test Blueprint of Pre-Twitter, Twitter, and Post-Twitter Vignette Survey**

|  | Learning objective #1 | | | Learning objective #2 | | | Learning objective #3 | | | Learning objective #4 | | |
| --- | --- | --- | --- | --- | --- | --- | --- | --- | --- | --- | --- | --- |
| **Remember**  (retrieve knowledge from long term memory) |  |  |  | 9a | 7b | 9c | 3a | 1b, 5b | 3c | 3a | 1b | 3c |
| **Understand**  (construct meaning from messages, images) |  |  |  | 8a |  | 8c |  |  |  |  |  |  |
| **Apply** (use the knowledge in a given situation) | 1a | 8b, 9b | 1c | 6a | 6b | 6c | 6a | 11b | 6c | 10a |  | 10c |
| **Analyze** (break material into parts and how it all fits together) |  |  |  | 2a | 4b | 2c |  | 13b |  |  | 13b |  |
| **Evaluate** (make judgements based on criteria/standards through critiquing) |  |  |  | 4a, 7a | 10b, 14b, 15b, 16b | 4c,  7c | 4a, 5a | 2b, 3b, 10b, 12b, 15b, 16b | 4c, 5c | 4a, 5a | 2b, 3b, 10b, 12b, 15b | 4c, 5c |
| **Create** (put elements together to create a whole, or reorganize into new structures) |  |  |  |  |  |  |  |  |  |  |  |  |
| **Total questions** | 1 | 2 | 1 | 6 | 7 | 6 | 4 | 10 | 4 | 4 | 7 | 4 |

Learning objective #1: Identify one cause of false positive of BDG testing

Learning objective #2: Distinguish at least one risk factor for each of the following fungal infections

Learning objective #3: Identify one appropriate indication for BDG testing

Learning objective #4: Identify one appropriate indication for GM testing

a- pre-Twitter vignette survey

b- Twitter vignette

c- post-Twitter vignette survey

**Supplement B: Pre- and post-Twitter Vignette Surveys**

Pre survey

Q1. What is your current position?

- Housestaff PGY-1 (1)
- Housestaff PGY-2 (2)
- Housestaff PGY-3 (3)
- Housestaff PGY-4 (4)
- Hospitalist (6)
- Advanced Practice Provider (7)
- ICU Attending (8)

Q2. At which institution do you primarily practice?

- University of Arizona (1)
- Columbia University Irving Medical Center (2)
- Johns Hopkins Hospital (3)
- Johns Hopkins Bayview Medical Center (4)

Q3. For how many patients with each of the following infections did you directly provide care in the past 3 months?

|  | 0 (1) | 1-3 (2) | 4-6 (3) | 7-9 (4) | 10 or greater (5) |
| --- | --- | --- | --- | --- | --- |
| Aspergillosis (1) |  |  |  |  |  |
| Candidemia (2) |  |  |  |  |  |
| Coccidioidomycosis (3) |  |  |  |  |  |
| Cryptococcosis (4) |  |  |  |  |  |
| Histoplasmosis (5) |  |  |  |  |  |
| Pneumocystis jirovecii pneumonia (6) |  |  |  |  |  |

Q4. In the past year, how many hours of didactic lecture have you received regarding fungal infections and diagnostics?

- 0 (1)
- 1 hour or less (2)
- 2-3 (3)
- 4-5 (4)
- >5 (5)

Q5. How would you rate your level of confidence in your ability to NAME the following?

|  | Very unconfident (1) | Unconfident (2) | Neither confident nor unconfident (3) | Confident (4) | Very confident (5) |
| --- | --- | --- | --- | --- | --- |
| risk factors for invasive fungal infections (1) |  |  |  |  |  |
| indications for a galactomannan test (2) |  |  |  |  |  |
| indications for a Beta-d-glucan test (3) |  |  |  |  |  |
| reasons for a false-positive Beta-d-glucan test (4) |  |  |  |  |  |

Q6. How important is it for you to have knowledge of the following?

|  | Very unimportant (1) | Unimportant (2) | Neither important nor unimportant (3) | Important (4) | Very important (5) |
| --- | --- | --- | --- | --- | --- |
| risk factors for invasive fungal infections (1) |  |  |  |  |  |
| test characteristics of Beta-d-glucan (2) |  |  |  |  |  |
| test characteristics of galactomannan (3) |  |  |  |  |  |

Q7. Which of the following methods have you used to learn about (select all that apply)?

|  |  | | | | | | | |  |
| --- | --- | --- | --- | --- | --- | --- | --- | --- | --- |
|  | Textbook/UpToDate (1) | Primary literature (2) | Didactic lecture (3) | Small group session(s) (4) | Online Module(s) (5) | Twitter case-based questions (6) | Pocket card (7) | None of these (8) | Other (1) |
| risk factors for invasive fungal infections (1) |  |  |  |  |  |  |  |  |  |
| test characteristics of Beta-d-glucan (2) |  |  |  |  |  |  |  |  |  |
| test characteristics of galactomannan (3) |  |  |  |  |  |  |  |  |  |

Q8. Which teaching method would you prefer for learning the following topics (please rank your top 3 options 1-3, with "1" being the most preferred)?

|  |  | | | | | | |  |
| --- | --- | --- | --- | --- | --- | --- | --- | --- |
|  | Textbook/ UpToDate (1) | Primary literature (2) | Didactic lecture (3) | Small group session(s) (4) | Online Module(s) (5) | Twitter case-based questions (6) | Pocket card (7) | Other (1) |
| risk factors for invasive fungal infections (1) |  |  |  |  |  |  |  |  |
| test characteristics of Beta-d-glucan (2) |  |  |  |  |  |  |  |  |
| test characteristics of galactomannan (3) |  |  |  |  |  |  |  |  |

Q9. How often do you use Twitter?

- Never (1)
- Once a month (2)
- Once a week (3)
- Three times a week (4)
- Daily (5)

Q10. If you do use Twitter, how effective is Twitter as a source of medical information?

- Very ineffective (1)
- Ineffective (2)
- Neither effective nor ineffective (3)
- Effective (4)
- Very effective (5)
- NA (6)

Q11. If you were to participate in a fungal diagnostics curriculum that had weekly Twitter questions to help with your learning, how many times a week would you be willing to answer questions?

- Zero (1)
- One (2)
- Two (3)
- Three (4)
- Daily (M-F) (5)
- Other (6) ________________________________________________

1a) 40M with PMHx of hypertension and ESRD on dialysis presents to the ED with fevers and abdominal pain for 3 days. Vitals: T 38.5°C, BP 80/40, HR 110, RR 18, O2 100% on room air. He received lactated ringers and albumin. Exam is unremarkable. Labs are notable for a WBC 11,000. CT chest/abdomen/pelvis shows atelectasis and gut wall edema in the large bowel. Blood cultures are sent and pending. Patient is started on ceftriaxone and albumin with dramatic improvement in fevers and blood pressure. 3 days later, someone orders a serum beta-d-glucan and results at 110 (positive >80). What is the most likely cause of this positive beta-d-glucan?

1. Pulmonary aspergillosis
2. **False + from albumin**
3. False + from ceftriaxone
4. *Candida* peritonitis

2a) 63M with acute myeloid leukemia (AML) s/p BMT 8 months ago presents to clinic with 4 weeks of fevers and cough. He has not been taking prophylactic antimicrobials for 6 weeks. Vitals- T 38.7°C, HR 105, BP 110/75, RR 16, O2 96% on room air. Labs notable for WBC 1,700 cells/mm^3^ (neutrophil count 400), hemoglobin 7.1 g/dL, platelets 45,000/mm^3^ (normal 8 weeks ago). CT chest notable for new 1cm right upper lobe nodule and 1cm left lower lobe nodule. There is suspicion for AML relapse. Serologic work-up is notable for beta-d-glucan of 60 (positive ≥ 80) and galactomannan of 1.5 (positive ≥ 0.5). Which of the following is the most likely diagnosis?

1. **Pulmonary aspergillosis**
2. Pulmonary mucormycosis
3. Pulmonary cryptococcosis
4. Pulmonary candidiasis

3a) 55F with rheumatoid arthritis presents to rheumatology clinic for consideration of advanced therapies. She has failed numerous regimens and the rheumatologist is considering rituximab. Which of the following tests should be ordered before starting therapy?

1. Serum beta-d-glucan
2. Serum galactomannan
3. **Hepatitis B core antibody**
4. Serum crypto antigen

4a) 56F with PMHx alcoholic cirrhosis, presents to the ED with dizziness and decreased urine output. She is found to be hypotensive and transferred to the ICU. She is started on vasopressors and CVVHD via central lines and is breathing comfortably on room air. Due to concern for spontaneous bacterial peritonitis and inability to perform a paracentesis, she is started on ceftriaxone. She remains critically ill in the ICU for 7 days with the 2 central lines still in place. On Day 8, she is febrile and develops rigors. In addition to blood cultures, which of the following is the next best test to order?

1. Lactate dehydrogenase
2. Serum galactomannan
3. **Serum beta-d-glucan**
4. Fungal blood culture

5a) 60F with COPD from Russia (never left the country) presents with shortness of breath secondary to a COPD exacerbation from running out of her inhalers. A CT chest shows a 5mm right upper lobe nodule. Which of the following is the next best test for further evaluation of the nodule?

1. Serum beta-d-glucan
2. Serum galactomannan
3. Blasto serum antibody
4. **Sputum AFB stain and culture**

6a) 75F with lupus (well-controlled), hypertension, and recent diagnosis of HIV (viral load 30,000, CD4 count 57) presents to ED in winter with 4 weeks of progressive shortness of breath and cough. Vitals: T 38.5°C, HR 105, BP 105/80, RR 20, O2 94% on 3L. Exam is notable for crackles and rhonchi throughout both lungs. Labs notable for WBC 2,600 (PMN 21%, lymph 44%), Hb 7.5 g/dL, platelet 70,000. CXR shows diffuse opacities. You have suspicion for *Pneumocystis jirovecii* pneumonia. Which of the following is the best non-invasive test to order to help confirm the diagnosis?

1. Lactate dehydrogenase
2. Serum galactomannan
3. Serum *Pneumocystis jirovecii* PCR
4. **Serum beta-d-glucan**

7a) 45M with PMHx COPD presents with 5 days of subjective fevers, shortness of breath, and cough. Vitals- T 38.2°C, HR 112, BP 100/75, RR 22, O2 93% on 100% non-rebreather. Exam is notable for diffuse crackles bilaterally. CT chest demonstrates emphysematous lungs and ground glass opacities bilaterally. Further evaluation is significant for +COVID-19 NAT respiratory swab, sputum and blood cultures are negative. After initiation of remdesivir, dexamethasone, and empiric ceftriaxone plus doxycycline, he defervesces. 3 weeks into his hospital course, he has fevers and worsening shortness of breath. Repeat CT chest shows worsening ground glass opacities and new pulmonary nodules. Respiratory viral panel, COVID-19 NAT, and blood cultures are negative. Which of the following is the most likely cause of the new nodules?

1. **Aspergillosis**
2. COVID-19
3. CMV pneumonitis
4. Candidiasis

8a) 45M with cirrhosis and diabetes presents with altered mental status and abdominal pain. On exam, he is hypoxic with abdominal distention. A diagnostic paracentesis reveals 260 neutrophils. He is started on ceftriaxone and albumin for spontaneous bacterial peritonitis. Which of the following additional factors puts the patient at highest risk for developing invasive candidiasis?

1. Knee surgery
2. Working at nursing home
3. **Central line for TPN**
4. Hypertension

9a) 55F with COPD presents with 3 months of shortness of breath and fatigue. She lives in Canada and loves to garden. After evaluation with her primary medical doctor (PMD), the PMD is concerned that she may have a hematologic malignancy. Which of the following puts the patient at HIGHEST risk for developing active invasive aspergillosis?

1. **Hematologic malignancy**
2. From Canada
3. Gardening
4. COPD

10a) 40F with cystic fibrosis s/p bilateral lung transplant 2 months ago presents with shortness of breath and worsening pulmonary function tests (PFTs) in clinic. She has been adherent to her medications that include tacrolimus, mycophenolate, prednisone, valganciclovir, clotrimazole-troches, and trimethoprim-sulfamethoxazole. Vitals- T36°C, HR 90, BP 110/75, RR 18, O2 94% on 2L. Exam notable for bilateral crackles. Her CBC and CMP are normal. A CT chest shows a new 2cm right upper lobe cavitary lesion and patchy ground glass opacities bilaterally. Her blood and sputum cultures are negative. Which of these diagnostics tests would be most likely to yield a diagnosis?

1. **BAL galactomannan**
2. Mycobacterial blood culture
3. Serum galactomannan
4. Histoplasma serum antibody

Post survey

Q1. What is your current position?

- Housestaff PGY-1 (1)
- Housestaff PGY-2 (2)
- Housestaff PGY-3 (3)
- Housestaff PGY-4 (4)
- Hospitalist (6)
- Advanced Practice Provider (7)
- ICU attending (8)

Q2. At which institution do you primarily practice?

- University of Arizona (1)
- Columbia University Irving Medical Center (2)
- Johns Hopkins Hospital (3)
- Johns Hopkins Bayview Medical Center (4)

Q3. For how many patients with each of the following infections did you directly provide care in the past 3 months?

|  | 0 (1) | 1-3 (2) | 4-6 (5) | 7-9 (7) | 10 or greater (8) |
| --- | --- | --- | --- | --- | --- |
| Aspergillosis (1) |  |  |  |  |  |
| Candidemia (2) |  |  |  |  |  |
| Coccidioidomycosis (3) |  |  |  |  |  |
| Cryptococcosis (4) |  |  |  |  |  |
| Histoplasmosis (5) |  |  |  |  |  |
| Pneumocystis jiroveci pneumonia (6) |  |  |  |  |  |

Q4. In the past year, how many hours of didactic lecture have you received regarding fungal infections and diagnostics?

- 0 (1)
- 1 hour or less (2)
- 2-3 (3)
- 4-5 (4)
- >5 (5)

Q5. How would you rate your level of confidence in your ability to NAME the following?

|  | Very unconfident (1) | Unconfident (2) | Neither confident nor unconfident (3) | Confident (4) | Very confident (5) |
| --- | --- | --- | --- | --- | --- |
| risk factors for invasive fungal infections (1) |  |  |  |  |  |
| indications for a galactomannan test (2) |  |  |  |  |  |
| indications for a Beta-d-glucan test (3) |  |  |  |  |  |
| reasons for a false-positive Beta-d-glucan test (4) |  |  |  |  |  |

Q6. How important is it for you to have knowledge of the following?

|  | Very unimportant (1) | Unimportant (2) | Neither important nor unimportant (3) | Important (4) | Very important (5) |
| --- | --- | --- | --- | --- | --- |
| risk factors for invasive fungal infections (1) |  |  |  |  |  |
| test characteristics of Beta-d-glucan (2) |  |  |  |  |  |
| test characteristics of galactomannan (3) |  |  |  |  |  |

Q7. Which of the following methods have you used to learn about (select all that apply)?

|  |  | | | | | | | |  |
| --- | --- | --- | --- | --- | --- | --- | --- | --- | --- |
|  | Textbook/UpToDate (1) | Primary literature (2) | Didactic lecture (3) | Small group session(s) (4) | Online Module(s) (5) | Twitter case-based questions (6) | Pocket card (7) | None of these (8) | Other (1) |
| risk factors for invasive fungal infections (1) |  |  |  |  |  |  |  |  |  |
| test characteristics of Beta-d-glucan (2) |  |  |  |  |  |  |  |  |  |
| test characteristics of galactomannan (3) |  |  |  |  |  |  |  |  |  |

Q8. Which teaching method would you prefer for learning the following topics (please rank your top 3 options 1-3, with "1" being the most preferred)?

|  |  | | | | | | |  |
| --- | --- | --- | --- | --- | --- | --- | --- | --- |
|  | Textbook/ UpToDate (1) | Primary literature (2) | Didactic lecture (3) | Small group session(s) (4) | Online Module(s) (5) | Twitter case-based questions (6) | Pocket card (7) | Other (1) |
| risk factors for invasive fungal infections (1) |  |  |  |  |  |  |  |  |
| test characteristics of Beta-d-glucan (2) |  |  |  |  |  |  |  |  |
| test characteristics of galactomannan (3) |  |  |  |  |  |  |  |  |

Q9. What percent of the Twitter-based questions did you complete?

- 0 (1)
- 1-20% (2)
- 21-40% (3)
- 41-60% (4)
- 61-80% (5)
- 81-100% (6)

Q10. How satisfied are you with the quality of the Twitter case-based curriculum?

- Very unsatisfied (1)
- Unsatisfied (2)
- Neither unsatisfied nor satisfied (3)
- Satisfied (4)
- Very satisfied (5)
- NA (6)

Q11. On average, how would you rate the difficulty of the Twitter case-based questions?

- Very easy (1)
- Easy (2)
- Neither easy nor difficult (3)
- Difficult (4)
- Very difficult (5)
- NA (6)

Q12. On average, how would you rate the clarity of the Twitter case-based questions?

- Very unclear (1)
- Unclear (2)
- Neither unclear nor clear (3)
- Clear (4)
- Very clear (5)
- NA (6)

Q13. On average, how would you rate the clarity of the "Tweetorials" (explanations following the questions)?

- Very unclear (1)
- Unclear (2)
- Neither unclear nor clear (3)
- Clear (4)
- Very clear (5)
- NA (6)

Q14. On average, how would you rate the length of "Tweetorials" (explanations following the questions)?

- Too short (1)
- Short (2)
- Just right (3)
- Long (4)
- Very long (5)
- NA (6)

Q15. On average, how informative were the "Tweetorials" (explanations following the questions)?

- Very uninformative (1)
- Uninformative (2)
- Neither uninformative nor informative (3)
- Informative (4)
- Very informative (5)
- NA (6)

Q16. Would you participate in a Twitter case-based curriculum again?

- Yes (1)
- No (2)

Q17. What were the strengths of this Twitter case-based curriculum?

Q18. How could this Twitter case-based curriculum be improved?

1b) 45F with a recent diagnosis of myasthenia gravis 2 weeks ago s/p plasmapheresis & IVIg presents to the ED with fevers and chills, but no shortness of breath or cough. She was found to have MRSA bacteremia and started on vancomycin. 3 days into therapy for the MRSA bacteremia, she continued to have fevers. In addition to blood cultures, the primary team also ordered a serum beta-d-glucan, which resulted as 350 (positive ≥ 80 pg/mL). What is the most likely cause of this elevated beta-d-glucan?

1. MRSA bacteremia
2. **IVIg**
3. Vancomycin
4. Myasthenia gravis

2b) 62F with history of myelodysplastic syndrome s/p BMT presents to clinic for a follow-up visit. She is taking voriconazole, moxifloxacin, and valacyclovir for prophylaxis. She is also taking prednisone 40mg daily for skin graft versus host disease. Vitals are normal and stable. Physical exam is notable for an erythematous, macular, morbilliform eruption on the face, chest, and back. Labs are notable WBC 1,800 cells/mm^3^ (neutrophil count 300), hemoglobin 7.6 g/dL, platelets 55,000/mm^3^. Which of the following features increase the risk of developing pulmonary aspergillosis?

1. CD4^+^ count 250 cells/µL
2. **Prednisone 40mg/d x 4 weeks**
3. DM w/ HbA1c 7% 2 weeks ago
4. Central venous catheter

3b) 55M with Crohn’s disease presents to GI clinic for consideration of advanced therapies. He has failed other regimens and the GI team is considering infliximab. Which of the following tests should be ordered before starting therapy?

**A) TB Interferon-gamma release assay**

B) Serum beta-d-glucan

C) Serum galactomannan

D) Serum crypto antigen

4b) 77F with history of cirrhosis and uncontrolled DM2 has been in the ICU for management of sepsis requiring vasopressors and CVVHD via a triple lumen catheter and dialysis catheter. She is receiving TPN. She has been on vancomycin and piperacillin-tazobactam for 7 days. While on antibiotics, she develops fevers and increasing vasopressor requirements. Her CXR is clear, and the UA is unremarkable. In addition to blood cultures, which of the following is the next best test to order?

1. CMV PCR
2. **Serum beta-d-glucan**
3. Fungal blood culture
4. Lactate dehydrogenase

5b) 70M with HTN presents to the ED with shortness of breath and cough for 3 days. Vitals- T 38.3°C, HR 110, BP 110/75, RR 18, O2 94% on 2L. Exam is significant for crackles at right lung base. Labs notable for WBC 12,000 cells/mm^3^. CXR shows a right lower lobe opacity. Which of the following diagnostics tests should be ordered next?

1. **Sputum cultures**
2. Cocci serology
3. Serum galactomannan
4. Serum beta-d-glucan

6b) 40M with HTN and HIV (viral load 100,000, CD4 count 100) presents to the ED with 4 weeks of progressive shortness of breath and cough. Vitals: T 38.4°C, HR 110, BP 110/80, RR 20, O2 95% on room air, but desats to 90% with ambulation. Exam is notable for crackles and rhonchi throughout both lungs. Labs notable for WBC 2,600 (PMN 25%, lymph 50%), Hb 8 g/dL, platelet 80,000. CXR shows diffuse opacities. Blood and sputum cultures are negative. You have suspicion for *Pneumocystis jirovecii* pneumonia. Which of the following is the best non-invasive test to order to help confirm the diagnosis?

1. Lactate dehydrogenase
2. Serum galactomannan
3. **Serum beta-d-glucan**
4. Fungal blood culture

7b) 45F presents with 5 days of subjective fevers, shortness of breath, and cough. Vitals- T 38.3°C, HR 110, BP 110/70, RR 22, O2 95% on 6L and is quickly intubated. Exam is notable for diffuse crackles bilaterally. CT chest demonstrates ground glass opacities bilaterally. Further evaluation is significant for +COVID-19 NAT respiratory swab; sputum and blood cultures are negative. After initiation of remdesivir, dexamethasone, and empiric ceftriaxone plus doxycycline, she defervesces. 3 weeks into her hospital course, she has fevers and worsening shortness of breath. Repeat CT chest shows worsening ground glass opacities and new pulmonary nodules. Which of the following is the most likely cause of the new nodules?

1. **Aspergillosis**
2. CMV pneumonitis
3. COVID-19
4. Candidiasis

8b) 65F with cirrhosis and DM2 has been in the ICU for management of sepsis requiring vasopressors via a triple lumen catheter. She has been on vancomycin and piperacillin-tazobactam for several days. While in the ICU, she develops acute abdominal distention, and an upright abdominal X-ray shows free air under the diaphragm. The patient is at highest risk for developing which of the following infections?

1. Aspergillosis
2. Histoplasmosis
3. Mucormycosis
4. **Candidiasis**

9b) 30F presents with 5 days of fevers and sore throat. 8 weeks ago, she was diagnosed with Graves’ disease and started on methimazole. She also takes inhaled budesonide for her asthma. She works as a construction worker. Vitals- T 38.5°C, HR 105, BP 110/70, RR 18, O2 95% on room air. Labs notable for WBC 1,000 cells/mm^3^ (neutrophil count 400). Which of the following puts the patient at HIGHEST risk for developing an invasive aspergillosis?

1. Inhaled budesonide
2. **Neutropenia**
3. Graves’ disease
4. Construction work

10b) 19M with acute myeloid leukemia (AML) s/p BMT 10 months ago presents to clinic with 3 weeks of fevers and cough. He has not been taking prophylactic antimicrobials for 8 weeks. Vitals- T 38.6°C, HR 100, BP 115/75, RR 16, O2 97% on room air. Labs notable for WBC 1,500 cells/mm^3^ (neutrophil count 600), hemoglobin 7.7 g/dL, platelets 35,000/mm^3^ (normal 8 weeks ago). CT chest notable for new 1cm right lower lobe nodule and 1cm left upper lobe nodule. There is suspicion for AML relapse. Given the new pulmonary nodules and neutropenia, there is concern for pulmonary aspergillosis. Which of the following would be most helpful in the diagnosis of pulmonary aspergillosis?

1. Serum beta-d-glucan
2. Serum galactomannan
3. **BAL galactomannan**
4. Sputum culture
